# Supplementary material for: Accumulation of Lymphoid Progenitors with Defective B Cell Differentiation and of Putative Natural Killer Progenitors in Aging Human Bone Marrow
Source: Int J Mol Sci. 2025 Oct 28;26(21):10467. doi: 10.3390/ijms262110467 (PMC12609476; doi:10.3390/ijms262110467)
Supplement: Supplementary file 1 [file ijms-26-10467-s001.zip › ijms-3921408-supplementary.pdf]

Supplementary Figure S1

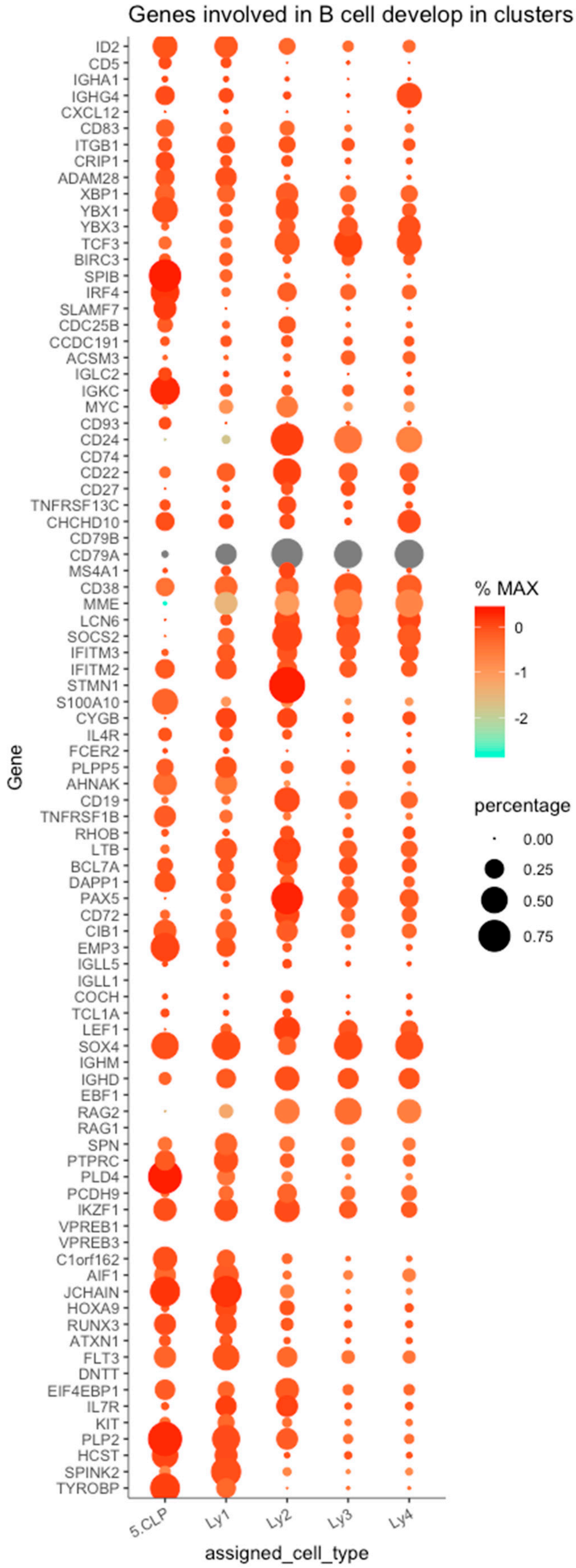

Supplementary Figure S1: Expression profile of genes involved in B cell development in each of the lymphoid sub-clusters

The expression profiles of the 90 genes involved in lymphoid development in the y-axis, are mapped to the lymphoid clusters in the x-axis.

Supplementary Figure S2

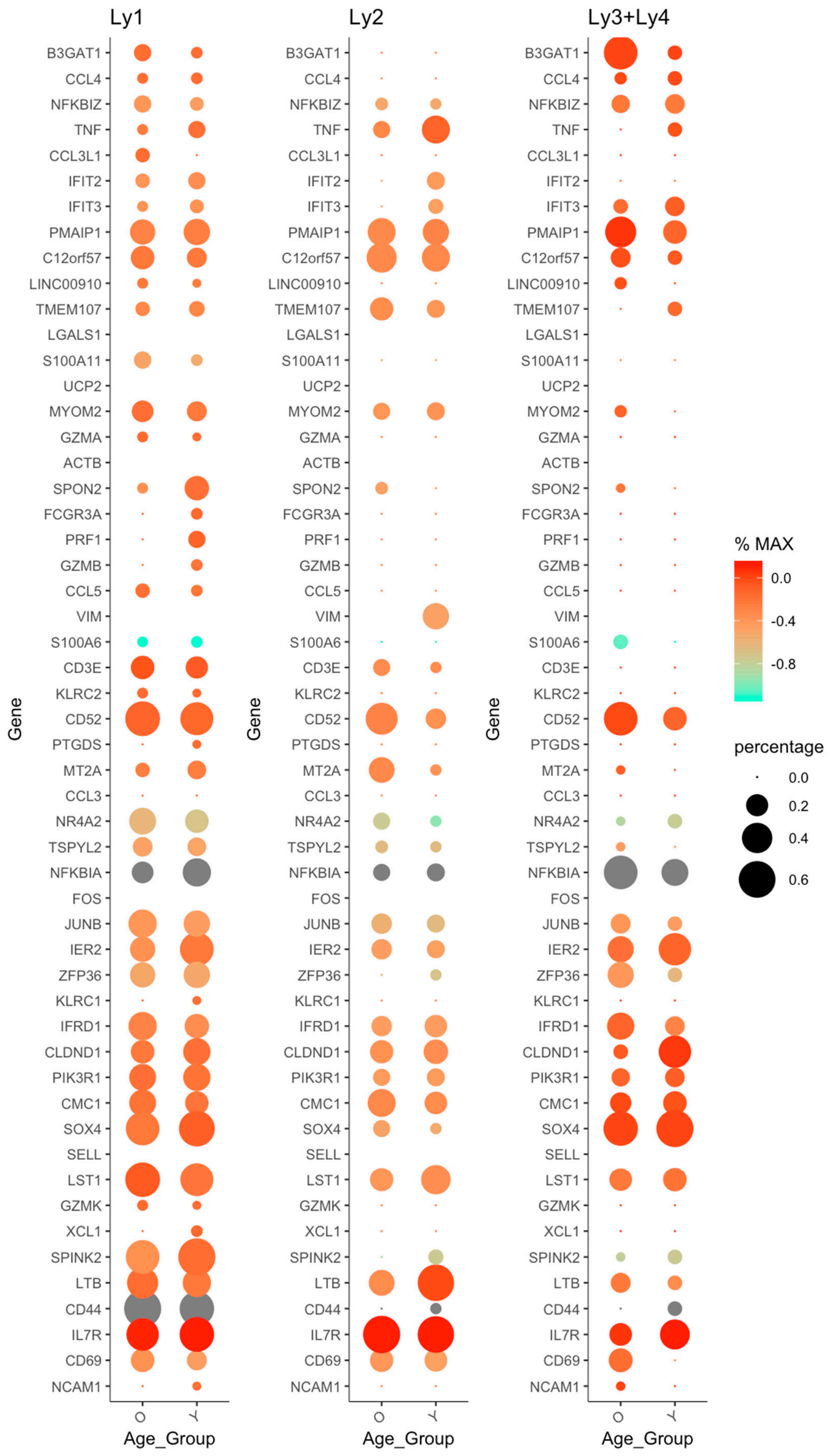

Supplementary Figure S2:
Transcriptome expression profiles of genes involved in development of NK progenitors and NK cells

The differential expression profiles of each of the genes involved in development of NK progenitors and NK cells between the two age groups are illustrated in the differentiation stages Ly1, Ly2 and Ly3+Ly4.
